# Exploring atherosclerosis imaging with contrast-enhanced MRI using PEGylated ultrasmall iron oxide nanoparticles

**DOI:** 10.3389/fbioe.2023.1279446

**Published:** 2023-09-20

**Authors:** Ruru Zhang, Kuan Lu, Li Xiao, Xuelan Hu, Wu Cai, Linjiang Liu, Yan Liu, Weihua Li, Hui Zhou, Zhiyuan Qian, Sixia Wang, Can Chen, Jianfeng Zeng, Mingyuan Gao

**Affiliations:** ^1^ Center for Molecular Imaging and Nuclear Medicine, State Key Laboratory of Radiation Medicine and Protection, School for Radiological and Interdisciplinary Sciences (RAD-X), Collaborative Innovation Center of Radiological Medicine of Jiangsu Higher Education Institutions, Soochow University, Suzhou, China; ^2^ The Second Affiliated Hospital of Soochow University, Suzhou, China; ^3^ Medical Imaging Department, Shenzhen Second People’s Hospital/The First Affiliated Hospital of Shenzhen University Health Science Center, Shenzhen, China

**Keywords:** atherosclerosis, ultrasmall iron oxide nanoparticles, magnetic resonance imaging, contrast agent, nanomedicine

## Abstract

Plaque rupture is a critical concern due to its potential for severe outcomes such as cerebral infarction and myocardial infarction, underscoring the urgency of noninvasive early diagnosis. Magnetic resonance imaging (MRI) has gained prominence in plaque imaging, leveraging its noninvasiveness, high spatial resolution, and lack of ionizing radiation. Ultrasmall iron oxides, when modified with polyethylene glycol, exhibit prolonged blood circulation and passive targeting toward plaque sites, rendering them conducive for MRI. In this study, we synthesized ultrasmall iron oxide nanoparticles of approximately 3 nm via high-temperature thermal decomposition. Subsequent surface modification facilitated the creation of a dual-modality magnetic resonance/fluorescence probe. Upon intravenous administration of the probes, MRI assessment of atherosclerotic plaques and diagnostic evaluation were conducted. The application of Flash-3D sequence imaging revealed vascular constriction at lesion sites, accompanied by a gradual signal amplification postprobe injection. T1-weighted imaging of the carotid artery unveiled a progressive signal ratio increase between plaques and controls within 72 h post-administration. Fluorescence imaging of isolated carotid arteries exhibited incremental lesion-to-control signal ratios. Additionally, T1 imaging of the aorta demonstrated an evolving signal enhancement over 48 h. Therefore, the ultrasmall iron oxide nanoparticles hold immense promise for early and noninvasive diagnosis of plaques, providing an avenue for dynamic evaluation over an extended time frame.

## 1 Introduction

Cardiovascular diseases (CVDs) remain a formidable global threat and are characterized by substantial morbidity, disability, and mortality rates. The World Health Organization (WHO) reports that CVDs claim approximately 17.9 million lives annually, accounting for a staggering 32% of global deaths (World Health Organisation, 2021). In China, the 2021 Cardiovascular Health and Diseases Report unveiled CVDs as a leading cause of mortality, with rural and urban populations experiencing 46.74% and 44.26% of the total fatalities, respectively (The Writing Committee of the Report on Cardiovascular Health and Diseases in China, 2022). Among the culprits in CVD development, atherosclerosis (AS) takes center stage, contributing to over 170,000 deaths annually across the nation (Zhao et al., 2019; Song et al., 2020). As a chronic inflammatory disease that endangers blood flow, atherosclerosis is characterized by the thickening of arterial wall and the formation of atherosclerotic plagues (Sanz and Fayad, 2008; Weber and Noels, 2011). Symptoms typically manifest in advanced stages, often resulting from the gradually narrowing of the artery lumens due to progressive plague growth or sudden rupture of plaques, leading to thrombosis (Zhong et al., 2022). Research underscores that nearly 70%–80% of major adverse cardiovascular events result from the rupture of unstable atherosclerotic plaques, an event that precipitates myocardial and cerebral infarctions (Roth et al., 2020). As a result, the early detection of atherosclerotic plaque formation is imperative.

The diagnosis of atherosclerotic plaques has seen a significant advancement in methodologies, each with its unique advantages and limitations (Chen et al., 2011; Chen and Wu, 2011). Starting with ultrasound (US), it offers the advantage of real-time imaging and noninvasiveness, enabling visualization of arterial walls and plaque morphology (Steinl and Kaufmann, 2015). However, its drawback lies in limited tissue penetration and resolution, making it less suitable for precise assessment. Computed tomography (CT) brings high spatial resolution to the table, allowing for detailed visualization of plaque composition and stenosis. However, its reliance on ionizing radiation poses risks, particularly for repeat scans (Sanz and Fayad, 2008). Nuclear medicine techniques such as positron emission tomography (PET) and single-photon emission computed tomography (SPECT) offer functional insights into plaque activity through radiotracers (Tarkin et al., 2014). These methods provide deep tissue penetration and can quantify biological processes (Joseph and Tawakol, 2016), but their spatial resolution remains a challenge, and the necessity for coregistration with anatomical images (e.g., CT) limits their stand-alone diagnostic potential. Considering that plaque is a chronic inflammatory disease influenced by various factors, the aforementioned diagnostic tools often fall short in terms of long-term evaluation. Magnetic resonance imaging (MRI) has emerged as a pivotal diagnostic tool for atherosclerotic plaques due to its multifaceted advantages. It offers high spatial resolution, excellent soft tissue contrast, and a prolonged imaging window without ionizing radiation. Moreover, MRI provides valuable insights into plaque composition, inflammation, and vascular remodeling (Fleg et al., 2012). Its ability to differentiate various plaque components, including lipid-rich cores and fibrous caps, contributes to comprehensive risk assessment (Wüst et al., 2019). In the context of atherosclerotic plaque diagnosis, MRI’s versatility and noninvasive nature grant it a distinct advantage, enabling thorough evaluation and risk stratification for improved patient management (Sosnovik et al., 2008).

Although MRI holds immense potential in diagnosing atherosclerotic plaques, it is important to acknowledge its limitations. MRI relies on intrinsic features such as proton density and relaxation time to distinguish normal and lesion tissues. However, this approach may not provide sufficient differentiation, prompting the need for contrast agents that alter water proton relaxation times, thus enhancing signal intensity and contrast (De Leon-Rodriguez et al., 2015; Jeong et al., 2018). Contrast agents are classified into T1, T2, and T1/T2 dual contrast agents based on their relaxation performances. T1 contrast agents shorten T1 relaxation, resulting in brighter images, while T2 contrast agents shorten T2 relaxation, yielding darker images. Currently, clinical contrast agents primarily include gadolinium-based agents, which excel in T1 imaging (Tweedle, 2020). Nevertheless, gadolinium-based agents carry inherent safety risks (Robert et al., 2020), especially due to their small molecular structure, resulting in short circulation times and a lack of specificity. These limitations somewhat restrict their applicability in diagnosing atherosclerotic plaques.

To overcome these limitations, nanotechnology has introduced nanoparticle-based MRI contrast agents, which hold significant promise for atherosclerotic plaque imaging (Dai et al., 2020). For instance, gadolinium-containing nanoparticles, such as ultrasmall NaGdF_4_ nanodots with PEG modification, were capable of atherosclerotic plaque imaging (Xing et al., 2014). In comparison to gadolinium-based agents, iron-based nanoparticles are expected to exhibit improved safety profiles (Chen et al., 2015). Among these, iron oxide nanoparticles, particularly Fe_3_O_4_ nanoparticles, have been extensively studied. Schmitz et al. and Ruehm et al. demonstrated that Fe_3_O_4_ nanoparticles are capable of being phagocytosed and taken up by macrophages at the plaque site in rabbit models of inherited or diet-induced atherosclerotic plaques (Schmitz et al., 2000; Ruehm et al., 2001). Kooi et al. reported the applicability of Fe_3_O_4_ nanoparticles for human plaque imaging (Trivedi et al., 2003). Typically ranging in size from 10 to 50 nm, these nanoparticles accumulate at rupture-prone sites, marking high-risk plaques (Ruehm et al., 2001; Trivedi et al., 2003; Trivedi et al., 2004; Hyafil et al., 2006; Boyer et al., 2010; Cortajarena et al., 2014; Stein-Merlob et al., 2017), and their extended circulation times contribute to an extended imaging window (Corot et al., 2003). Consequently, it is noteworthy that current imaging techniques often rely on negative T2-weighted imaging (Sadeghi et al., 2010; Li et al., 2019). However, to enhance contrast in regions of interest, the introduction of positive T1 contrast agents becomes pivotal. Given that the size of iron oxide nanoparticles significantly influences MRI effects, ultrasmall sub-5 nm iron oxide nanoparticles exhibit remarkable T1 effects, positioning them as an ideal choice for diagnosing atherosclerotic plaques (Usman et al., 2015; Chen et al., 2022).

In light of the aforementioned potential benefits of nanoparticle-based MRI contrast agents, the current study aimed to contribute to the field of atherosclerotic plaque imaging by focusing on the development of ultrasmall iron oxide nanoparticles as efficient T1 contrast agents. We first synthesized ultrasmall iron oxide nanoparticles with a core size of approximately 3 nm using a high-temperature thermal decomposition method. Building upon this synthesis, we designed a magnetic resonance/fluorescence dual-modality nanoprobe for enhanced imaging capabilities. This nanoprobe, consisting of ultrasmall iron oxide nanoparticles, exhibited both excellent T1 contrast enhancement potential and fluorescence imaging ability, making it a promising tool for atherosclerotic plaque diagnosis. Systematic *in vitro* and *in vivo* evaluations enabled us to demonstrate the nanoprobe’s ability to provide not only morphological assessment of plaque stenosis but also long-term dynamic monitoring and evaluation of aortic and carotid plaques.

## 2 Materials and methods

### 2.1 Materials

Ferric acetylacetonate (Fe (acac)3), oleic acid, oleamine, cyclohexane, tetrahydrofuran (THF), acetone, 2-aminoethanethiol, tris(2–carboxyethyl) phosphine, and Cy5.5-NHS were purchased from Shanghai Aladdin Biochemical Technology Co., Ltd. Polyethylene glycol (Mw ≈ 2000) functionalized with a diphosphate group at one end and a maleimide group at the other end, denoted as DP-PEG-Mal, was supplied by Suzhou Xinying Biomedical Technology Co., LTD. HEPES buffer was acquired from Beijing Solarbio Science & Technology Co., Ltd. DMEM and fetal bovine serum (FBS) were procured from HyClone.

### 2.2 Preparation of PEGylated Fe_3_O_4_ nanoparticles

The PEGylated Fe_3_O_4_ nanoparticles (Fe_3_O_4_-DP-PEG-Mal) were prepared following a modified procedure from a prior study (Zeng et al., 2014). In brief, hydrophobic Fe_3_O_4_ nanoparticles with a core size of 3 nm were synthesized. Subsequently, 10 mg of these hydrophobic Fe_3_O_4_ nanoparticles were dissolved in 3 mL of tetrahydrofuran (THF) containing 100 mg of DP-PEG-Mal. The reaction mixture was heated to 60°C and maintained at this temperature for 24 h with continuous stirring. After completion, the reaction solution was allowed to cool to room temperature. The resultant nanoparticles were then precipitated, washed with cyclohexane three times, and subsequently dried under vacuum at room temperature. The PEGylated Fe_3_O_4_ nanoparticles were dispersed in water at 4°C overnight. Finally, the purified Fe_3_O_4_ nanoparticles were obtained by subjecting them to ultrafiltration for three cycles using a 30 kDa MWCO centrifugal filter (Millipore YM-30) at 2,500 g.

### 2.3 Preparation of Cy5.5-modified Fe_3_O_4_ nanoparticles

The Cy5.5-modified Fe_3_O_4_ nanoparticles (Fe_3_O_4_-Cy) were obtained through the following steps. First, the amination of nanoparticles was accomplished using a click reaction. To elaborate, 2-aminoethanethiol (0.55 mg, 7.14 μmol) dissolved in water was mixed with an aqueous solution of tris(2–carboxyethyl) phosphine (6.58 mg, 22.96 μmol) and stirred for 5 min. After neutralizing the mixture with HEPES buffer (pH = 7.4, 1 M), it was added to the solution of PEGylated Fe_3_O_4_ nanoparticles (10 mg) and oscillated for 4 h. To eliminate excess 2-aminoethanethiol, the resulting solution was subjected to purification through ultrafiltration for three cycles using a 30 kDa MWCO centrifugal filter (Millipore YM-100). Subsequently, Cy5.5-NHS was coupled to the aminated nanoparticles. In brief, Cy5.5-NHS (0.5 mg, 0.71 μmol), dissolved in DMSO, was added to 10 mg of surface-aminated Fe_3_O_4_ nanoparticles and stirred overnight at room temperature. The resultant mixture was dialyzed for 72 h and ultrafiltered using 30 kDa ultrafiltration tubing to remove free Cy5.5-NHS and other reactants, yielding a solution of the Cy5.5-modified Fe_3_O_4_ nanoparticles.

### 2.4 Characterizations

The morphologies of the hydrophobic nanoparticles, Fe_3_O_4_-DP-PEG, and Fe_3_O_4_-Cy, were characterized using transmission electron microscopy (TEM, Talos F200S G2) with an acceleration voltage of 200 kV. The hydrodynamic size and zeta potential of the nanoparticles were measured using a Malvern Zetasizer Nano ZS90. The ultraviolet‒visible absorption spectrum was measured using a Shimadzu UV‒Vis spectrophotometer UV-3600 with quartz cuvettes. The fluorescence spectra were recorded using an Edinburgh FLS980 fluorescence spectrophotometer. The relativity measurements and *in vivo* magnetic resonance imaging were conducted using a 3 T animal MRI scanner (MRS 3000, MR Solution, Guidfore, United Kingdom). Fluorescence imaging was obtained using the IVIS Spectrum imaging system (PerkinElmer, Inc.).

### 2.5 Cell culture and cytotoxicity evaluation

RAW 264.7 cells and 3T3 cells were cultured in DMEM supplemented with 10% fetal bovine serum and 1% antibiotics (penicillin‒streptomycin) and maintained in an environment containing 5% CO_2_ at 37°C. Cell viability was assessed using CCK-8 assays. Briefly, 1 × 10^4^ cells/well were seeded into a 96-well plate and incubated overnight at 37°C under 5% CO_2_. After being washed twice with PBS, the nanoprobe solutions with iron concentrations ranging from 0 to 100 μg/mL were sequentially added, with three replicate wells set up for each concentration. After 24 h of incubation, the medium was replaced with DMEM containing 10% CCK-8 solution. After an additional 1.5 h, the absorbance at 450 nm for each well was measured using a multifunctional microplate reader, and the absorbance values were used to calculate the cell survival rate using the following formula: cell survival rate = (experimental wells—blank wells)/(control wells—blank wells) × 100%.

### 2.6 Animal models of atherosclerotic plaques

Male 6-week-old APOE^−/−^ mice were procured from Changzhou Cavins Laboratory Animal Co. Ltd. All animal experiments were conducted following the ethical guidelines and were approved by the Animal Ethics Committee of Soochow University Laboratory Animal Centre. The APOE^−/−^ mice were subjected to a high-fat diet (HFD, 15% fat, 1.25% cholesterol) for 2 weeks. Subsequently, a rigid polymer ketone perivascular cuff (Promolding, Netherlands) was placed around the right common carotid artery (Qiao et al., 2017). The mice continued on the HFD for an additional 6 weeks to establish the atherosclerotic plaque model.

### 2.7 MRI of carotid and aortic plaques

For the evaluation of carotid plaque morphology using MR Flash sequences, atherosclerotic mice were anesthetized with 5% isoflurane, and the mouse coil accompanying the MR system was utilized. After placing the mice in the animal bed, imaging was conducted at different time points both before and after intravenous injection of 200 μL Fe_3_O_4_-Cy nanoprobe (0, 0.5, 12 h), maintaining a consistent 2% isoflurane concentration throughout the scanning procedure. The Flash sequence parameters used for this purpose were as follows: TE = 4 ms, TR = 20 ms, averages = 6, FA = 30°, MTX = 256 × 256, FOV = 40 × 40 mm^2^, slice thickness = 0.8 mm, and gradient compensation scale = −10. Subsequently, the acquired cross-sectional images were reconstructed into 3D images using ImageJ software. The signal intensities at the plaque lesion site, the normal lumen, and the background were quantified utilizing the software of the imaging system. To assess the signal enhancement within the region of interest (ROI), the signal-to-noise ratio (SNR) values of the stenotic and normal vessels were calculated using the formula SNR = SI/σ, where SI represents the signal intensity within the ROI and σ is the standard deviation obtained from the background analysis of the MR images. The signal ratio before and after injection was determined using the contrast-to-noise ratio (CNR) formula: signal ratio = SNR post-injection/SNR pre-injection.

For T1-weighted imaging of carotid or aortic plaques, the Fast Spin Echo (FSE) sequence was employed. The parameters used for image acquisition were as follows: TE = 11 ms, TR = 720 ms, averages = 6, FA = 90°, MTX = 256 × 256, FOV = 40 × 40 mm^2^, and slice thickness = 0.8 mm. Subsequently, the MR signals originating from the plaque regions and the contralateral normal vascular regions of three mice were subjected to quantitative analysis using the software of the imaging system. This analysis allowed the extraction of signal values, which were then presented as the mean values along with their corresponding standard deviations (mean ± SD). The signal ratio, computed as the plaque signal on the induced side divided by the signal on the control side, was calculated based on these signal values.

### 2.8 Fluorescence imaging

To perform *in vitro* fluorescence imaging of nanoparticle samples, solutions containing the nanoparticles were meticulously prepared in 96-well plates and carefully positioned on an IVIS imaging platform. The excitation and emission wavelengths were specifically set to Ex = 660 nm and Em = 710 nm, respectively. For fluorescence imaging of isolated carotid artery tissues, common carotid artery tissues from both sides were extracted at various time points (12, 24, 48, 72 h) following the injection of Fe_3_O_4_-Cy solution via the tail vein. Black cardboard was utilized as the background during imaging, and specific IVIS parameters were configured as Ex = 660 nm and Em = 710 nm. Subsequently, the fluorescence signals of the ROIs located in the plaque region and the normal region of the control-side vessels were quantitatively analyzed.

### 2.9 H&E staining, Masson staining and Prussian blue staining

To validate the presence of atherosclerotic plaques, a comprehensive set of histological staining techniques was employed. For H&E staining of carotid artery tissues, bilateral carotid common artery samples were meticulously extracted at both the 4-week and 8-week time points. Following MRI imaging of the carotid artery, the harvested tissues underwent H&E staining. Furthermore, bilateral carotid artery tissues were subjected to H&E staining, Masson staining, and Prussian blue staining subsequent to T1-weighted imaging. The aortic artery tissues were also collected for H&E staining and Prussian blue staining after T1-weighted imaging. For the biosafety evaluation, major organs, including the heart, liver, spleen, lung, and kidney, were harvested for H&E staining analysis upon the injection of the Fe_3_O_4_-Cy nanoprobe. All collected tissues were meticulously fixed in paraformaldehyde to ensure optimal preservation of tissue morphology. Subsequently, the appropriate staining protocols were applied to the fixed tissues.

### 2.10 Statistical analysis

Continuous variables are presented as the mean ± standard deviation (SD). Comparative analyses between the two independent groups were conducted using Student’s *t*-test. Throughout the entirety of the experiment, two-sided tests were employed. Statistical significance was established at a threshold of *p* < 0.05.

## 3 Results and discussion

### 3.1 Synthesis and characterization of Fe_3_O_4_-Cy nanoparticles

**FIGURE 1:**
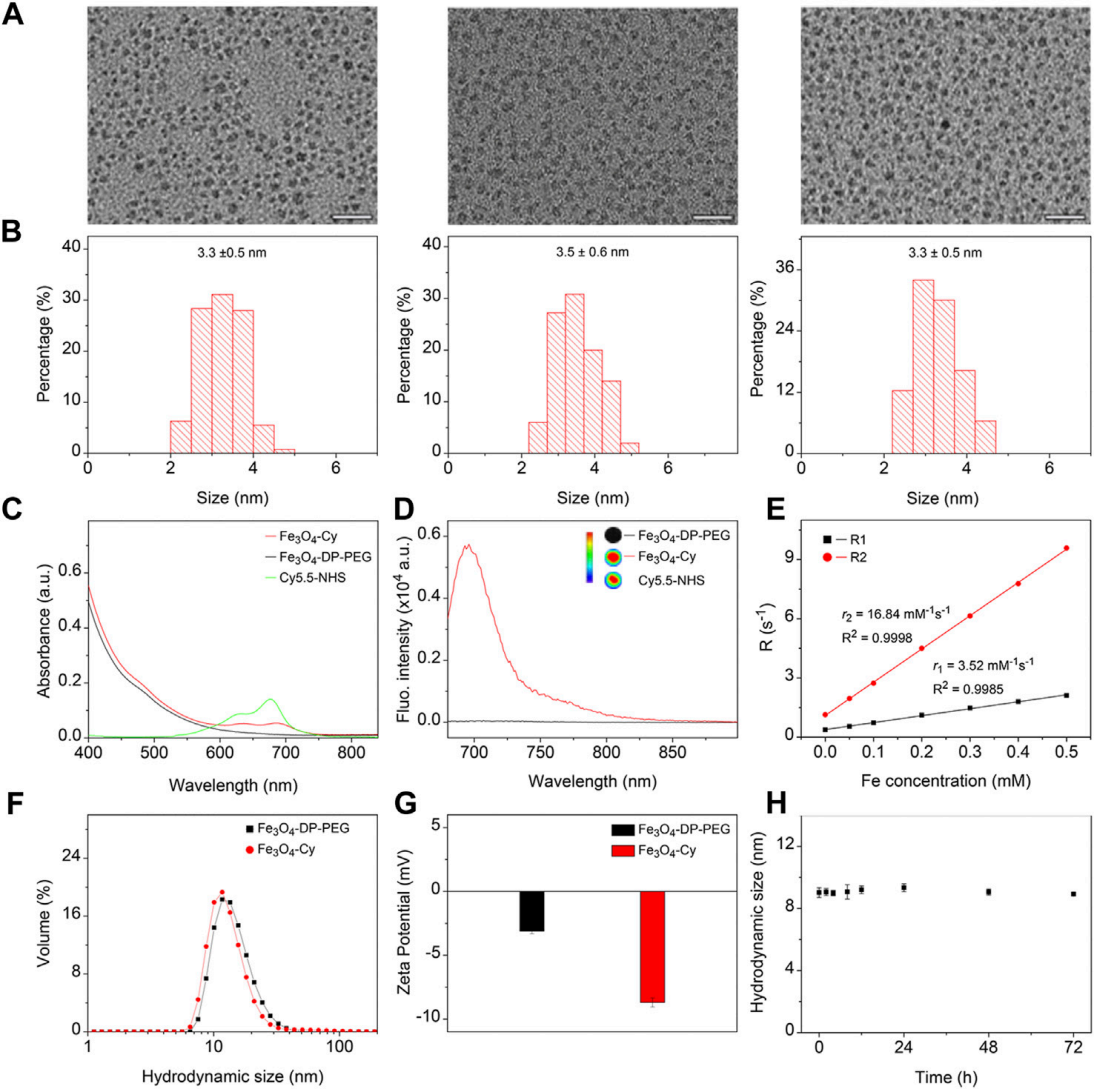
Synthesis and characterization of Fe_3_O_4_-Cy nanoparticles. **(A)** Representative TEM images of hydrophobic Fe_3_O_4_ nanoparticles, Fe_3_O_4_-DP-PEG, and Fe_3_O_4_-Cy, along with corresponding size histograms **(B)**; **(C)** UV absorption spectra of Fe_3_O_4_-DP-PEG, Cy5.5-NHS, and Fe_3_O_4_-Cy; **(D)** Fluorescence images of Fe_3_O_4_-DP-PEG, Cy5.5-NHS, and Fe_3_O_4_-Cy and fluorescence spectra of iron oxide nanoparticles before and after Cy5.5 modification; **(E)** Plots of R1 and R2 against Fe concentrations of Fe_3_O_4_-Cy, overlaid with linear fitting curves for extracting longitudinal and transverse relaxivities; **(F)** Hydrodynamic size of Fe_3_O_4_-DP-PEG and Fe_3_O_4_-Cy; **(G)** Zeta potential of Fe_3_O_4_-DP-PEG and Fe_3_O_4_-Cy; **(H)** Hydrodynamic size changes of Fe_3_O_4_-Cy incubated in 10% FBS for 72 h.

The hydrophobic Fe_3_O_4_ nanoparticles were synthesized via a thermal decomposition method. To confer hydrophilicity, polyethylene glycol (PEG) polymers with a diphosphate group at one end and a maleimide group at the other were utilized to replace the native organic ligands. For fluorescence imaging capability, the near-infrared dye Cy5.5-NHS was conjugated to Fe_3_O_4_ nanoparticles through an amidation reaction. The resulting nanoparticles were designated Fe_3_O_4_, Fe_3_O_4_-DP-PEG, and Fe_3_O_4_-Cy. Transmission electron microscopy (TEM) images depicted uniform nanoparticles with similar size distributions (Figure 1A). Analysis via ImageJ confirmed their monodispersity, with mean sizes of 3.3 ± 0.5, 3.5 ± 0.6, and 3.3 ± 0.5 nm, respectively (Figure 1B). These observations indicated that surface modification minimally affected nanoparticle morphology and size. The results indicated that the surface modification processes did not obviously alter the morphology and size distribution of the nanoparticles.

UV‒Vis spectrophotometry was employed to analyze the absorption spectra of Cy5.5-NHS, Fe_3_O_4_-DP-PEG, and Fe_3_O_4_-Cy (Figure 1C). While Fe_3_O_4_ nanoparticles displayed no distinct absorption peak, Cy5.5-NHS exhibited an absorption peak at 675 nm. Notably, after conjugation with Fe_3_O_4_-DP-PEG, the absorption peak redshifted to 682 nm, confirming successful coupling of the fluorescent dye to the nanoparticle surface. To evaluate the fluorescence imaging ability, fluorescence imaging of Cy5.5-NHS, Fe_3_O_4_-DP-PEG, and Fe_3_O_4_-Cy was conducted, illustrating robust signals from Fe_3_O_4_-Cy and Cy5.5-NHS, while Fe_3_O_4_ showed no signal. The fluorescence spectra of Fe_3_O_4_-DP-PEG and Fe_3_O_4_-Cy further validated their fluorescence imaging capability (Ex = 660 nm), transitioning from negligible to strong fluorescence intensity postcoupling (Figure 1D).

The relaxation rate measurements, crucial for MRI assessment, demonstrated that Fe_3_O_4_-Cy was an effective T1 contrast agent with a longitudinal relaxation rate (*r*_1_) of 3.52 mM-1 s-1, transverse relaxation rate (*r*_2_) of 16.84 mM^−1^ s^−1^, and *r*_2_/*r*_1_ ratio of 4.78 (Figure 1E). The corroborating *in vitro* MR images (Supplementary Figure S1) aligned with these results, demonstrating a direct relationship between Fe concentration and enhanced T1 contrast enhancement. Dynamic light scattering (DLS) assessments (Figures 1F, G) revealed hydrodynamic sizes of 14.8 nm (Fe_3_O_4_-DP-PEG) and 14.1 nm (Fe_3_O_4_-Cy), along with zeta potentials of −3.1 mV and −8.7 mV, respectively. These findings indicated minimal changes in hydrodynamic size and zeta potential after Cy5.5 modifications. Additionally, the hydrodynamic size of Fe_3_O_4_-Cy dispersed in a 10% FBS solution was tracked over time (Supplementary Figure S2), revealing negligible changes over 72 h (Figure 1H). This robust stability is imperative for the probe’s *in vivo* applications.

### 3.2 Cytotoxicity evaluation of the Fe_3_O_4_-Cy nanoprobe

A fundamental prerequisite for *in vivo* imaging applications is the comprehensive evaluation of probe biosafety. The cytotoxicity of a nanoprobe hinges not only on the inherent chemical composition of its core but also on the intricate interplay between surface physicochemical attributes and cellular interactions. Therefore, a meticulous assessment of Fe_3_O_4_-Cy cytotoxicity was conducted via cell viability measurements, employing the CCK-8 kit. As depicted in Supplementary Figure S3, the co-incubation of Fe_3_O_4_-Cy with RAW 264.7 and 3T3 cells exhibited no substantial decrease in viability across the 0–100 μg/mL Fe concentration range over a 24 h period. This compelling outcome underscores the low cytotoxicity exhibited by our nanoprobes, affirming their suitability for *in vivo* imaging experiments.

### 3.3 Verification of atherosclerotic plaques

Supplementary Figure S4A reveals the process of atherosclerotic plaque model construction, where plaques were formed by a combination of three distinct strategies: gene knockout, a high-fat diet regimen, and the strategic placement of a perivascular cuff to manipulate hemodynamic conditions. To meticulously track the progression of plaque formation, bilateral carotid artery tissues were meticulously harvested for H&E staining at two pivotal time points: 4 and 8 weeks following the inception of the modeling process. The outcomes, captured in Supplementary Figure S4B, vividly showcased the transformative changes occurring within the vascular environment. At the 4-week mark, a discernible thickening of the intima-media was observed on the experimental side in comparison to the control counterpart. Subsequently, at the 8-week juncture, a profound alteration in the arterial architecture emerged. Notably, the vessels on the experimental side exhibited remarkable stenosis, substantiating the progression of plaque development. Evident plaque formations within the blood vessels manifested as conglomerates of macrophages and foam cells encased by intricate collagen networks. These compelling observations collectively affirm the successful establishment of the atherosclerosis model over an 8-week duration, signifying the successful emulation of the targeted pathological condition.

### 3.4 *In vivo* flash sequence-based MRI for morphologic evaluation of plagues

**FIGURE 2:**
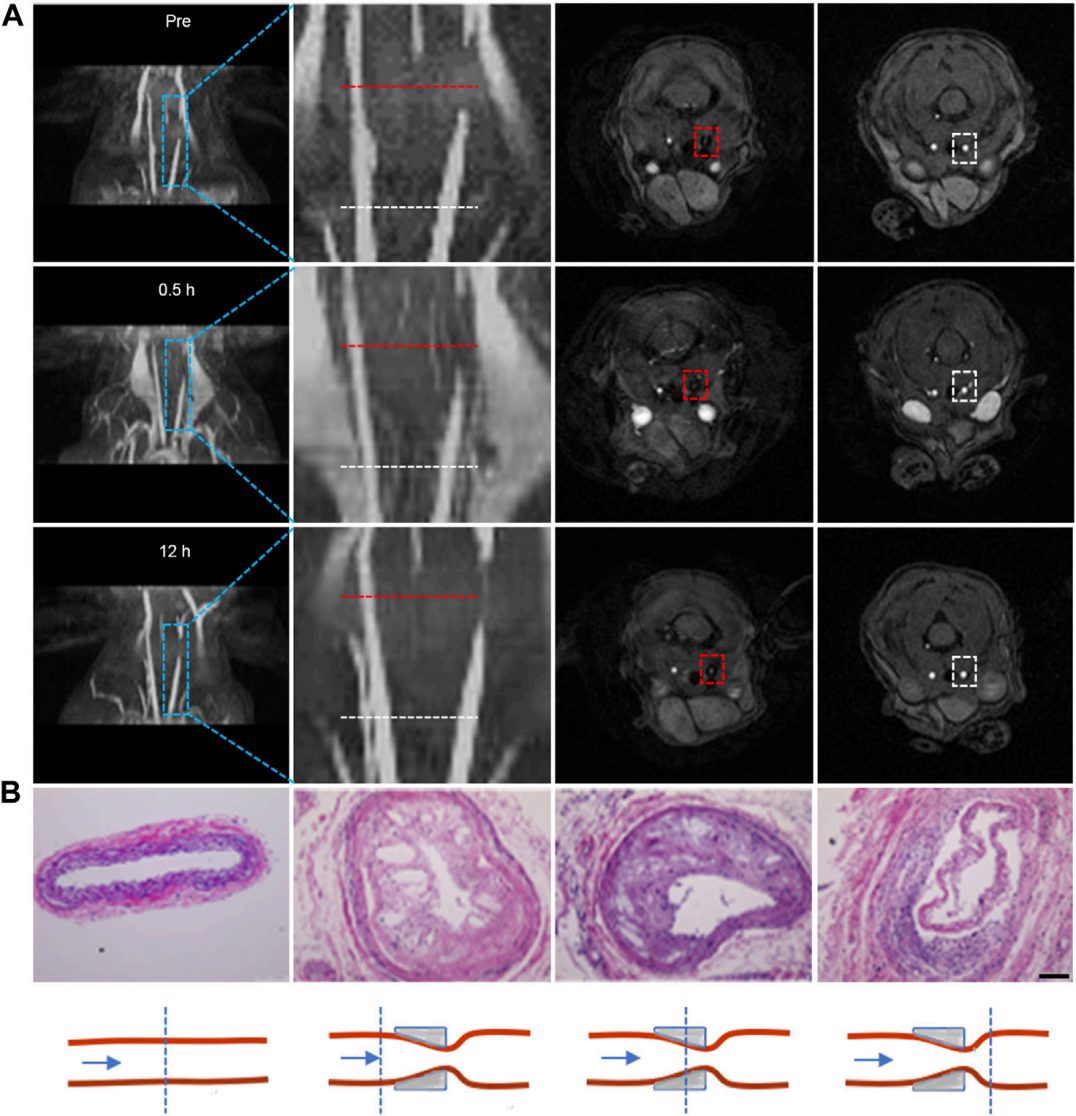
Flash sequence-based MRI imaging for morphologic evaluation of plagues. **(A)** 3D reconstructed images of the carotid artery at different time points before and after probe injection, along with corresponding 2D cross-sectional MRI images. The second column displays local magnifications of the first column; the third and fourth columns depict 2D cross-sectional MRI images corresponding to stenotic and normal proximal vessel ends, respectively. (Note: the red dotted line indicates lesion sites; the white dotted line indicates the normal lumen of the vessel proximal to the heart); **(B)** H&E staining of tissues at the lesion site and normal lumen of carotid tissues extracted from mice, accompanied by a schematic representation of the lesion site and normal lumen of carotid tissues. (Scale bar: 100 μm).

The presence of plaques within blood vessels inevitably induces alterations in vascular morphology. Hence, our initial focus was on evaluating the vessel morphology by introducing Fe_3_O_4_-Cy solution into the tail veins of atherosclerotic plaque model mice at a dose of 0.1 mmol/kg (corresponding to Fe concentration) and performing MRI at distinct time points before and after injection (0.5 h, 12 h) using a Flash sequence. Both 3D reconstructed images obtained through ImageJ and localized zoomed-in images illustrated a signal void at the site of plaques prior to probe injection, indicating the presence of narrowed vessels at the lesion sites. The signal at these defect sites exhibited enhancement at 0.5 h post-injection and further intensification at 12 h, indicative of the uptake of iron oxide nanoparticles at the plaque location (Figure 2A). Subsequent to MRI, tissue sections from three representative levels of the common carotid artery—encompassing the control side, both sides of the cannula, and the cannula site—were excised for H&E staining (Figure 2B). The outcomes demonstrated that the lumen of the control side retained its normalcy, displaying no discernible alterations in the intima. Conversely, the vessels on the afflicted side exhibited varied degrees of luminal constriction across all three levels, accompanied by intimal thickening and the emergence of numerous foam cells and cholesterol crystals. This further corroborated the progression of plaque formation on the diseased side. Quantitative analysis via MRI post-processing unveiled a substantial increase in the CNR at the lesion sites after injection compared to the CNR before injection, particularly when contrasted against the proximal vessels. This reinforced the notion that iron oxide nanoparticles were capable of accumulation at the plaque sites, leading to augmented vascular signals (Supplementary Figure S5). The collective findings unequivocally demonstrated the potency of iron oxide nanoparticles, in combination with Flash sequences, to effectively evaluate vascular morphology in the presence of plaques.

### 3.5 *In vivo* T1 MR imaging and *in vitro* fluorescence imaging of carotid plaques

**FIGURE 3:**
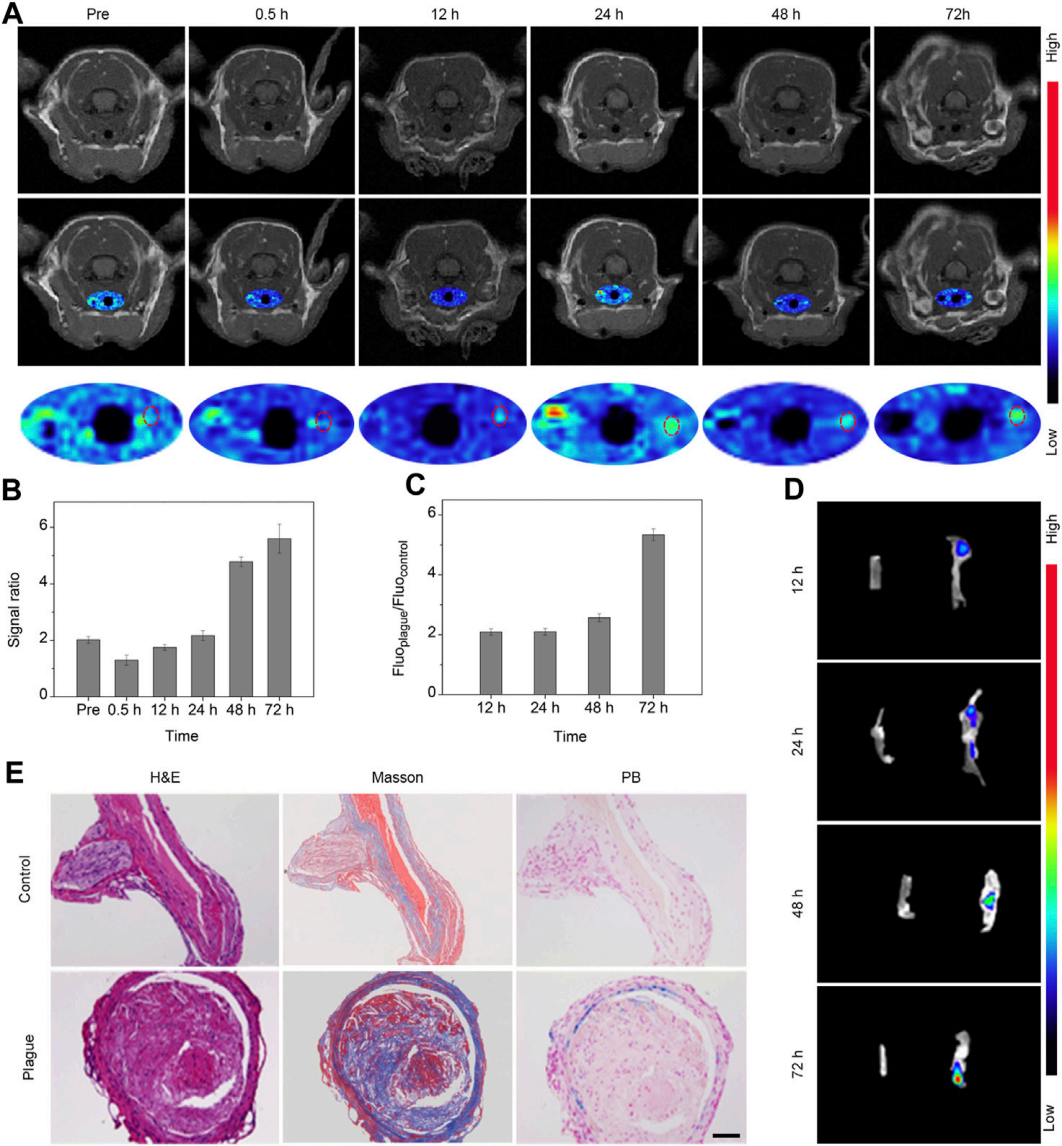
*In vivo* T1 MR imaging and *in vitro* fluorescence imaging of carotid plaques. **(A)** MR images and pseudocolor maps of mice captured at different time points post intravenous injection of probes. **(B)** Quantitative results (signal ratio between lesion sites and control sites) of T1 signal in the region of interest following intravenous probe injection; **(C)** Quantitative analysis of fluorescence intensity ratio within the region of interest; **(D)** Fluorescence image of bilateral carotid arteries extracted from mice; **(E)** H&E, Masson, and Prussian blue staining of bilateral carotid arteries extracted from mice 72 h after the injection of Fe_3_O_4_-Cy. (Scale bar: 100 μm).

To explore the extended behavior of plaques with enhanced resolution, we performed T1-weighted imaging via FSE sequences at various intervals prior to and following intravenous administration of the Fe_3_O_4_-Cy nanoprobe (0.5, 12, 24, 48, and 72 h). These images were subsequently subjected to pseudocolor processing. As shown in Figure 3A, signals within the right common carotid artery exhibited a gradual enhancement over time. Quantitative analysis of the vessel’s region of interest was conducted, revealing a progressive increase in the signal ratio between the plaque-bearing side and the control side, culminating in a peak value at 72 h post-injection (Figure 3B). These findings underscored the nanoprobe’s potential to enable continuous and dynamic assessment of atherosclerotic plaques.

To validate that the signal changes observed in the *in vivo* MRI were indeed attributable to the retention of iron oxide nanoparticles, bilateral common carotid artery tissues were harvested at various intervals post-injection for fluorescence imaging utilizing the IVIS system. Subsequent quantitative data analysis was carried out through post-processing with IVIS processing software (Figures 3C, D). The fluorescence images vividly portrayed a progressive rise in signal within the right vessel over time, in stark contrast to the negligible signal observed in the control site. This trend indicated the accumulation of the probe within the plaque region. Quantitative analysis of the data unveiled a gradual increase in the signal ratio between the plaque-bearing side and the control side, peaking at an approximate value of 5.7 at the 72 h time point, strongly suggesting enrichment of the probe within the plaque region.

Following imaging, the bilateral common carotid artery tissues were subjected to H&E staining, Masson staining, and Prussian blue staining (Figure 3E). The diseased side exhibited substantial plaques replete with abundant macrophages, foam cells, and fibrillogenesis in stark contrast to the normal vessels on the control side. Additionally, the plaque site exhibited a conspicuous deposit of blue substance, signifying a substantial accumulation of iron oxide nanoparticles. Further analysis of the staining results revealed that iron ions was deposited in smooth muscle cells, endothelial cells, and macrophage cells. A comprehensive review of the literature led to the conclusion that iron oxide nanoparticles enriched at the plague sites via the EPR effect and phagocytosis (Fleg et al., 2012). Collectively, these outcomes validated the presence of plaques at the imaged vessel site and the enrichment of Fe_3_O_4_ nanoparticles. This strongly supported the notion that the Fe_3_O_4_-Cy nanoprobe can effectively achieve sustained and dynamic assessment of atherosclerotic plaques.

### 3.6 *In vivo* T1 MR imaging of aortic plaques

**FIGURE 4:**
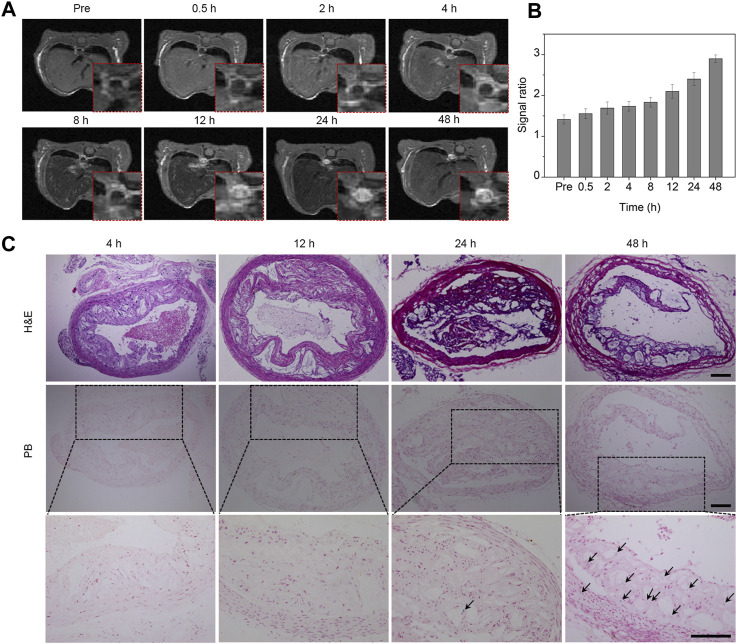
*In vivo* T1 MR imaging of aortic plaques. **(A)** MR images of the aorta captured at different time points following intravenous injection of the probe. The red dashed box in the bottom right corner represents partially enlarged images. **(B)** Quantitative analysis of the signal ratio between the plaque sites and muscle tissue. **(C)** H&E and Prussian blue staining of the aorta at various time points after probe injection. The bottom row consists of a series of magnified images corresponding to the middle row in panel **(C)** (Arrow, iron staining; scale bar, 100 μm).

The formation of atherosclerotic plaques is a common occurrence not only in the carotid artery but also in the aorta. To validate the imaging prowess of Fe_3_O_4_ nanoparticles for assessing plaques across different locations, we investigated the imaging characteristics of Fe_3_O_4_ nanoparticles in an aortic plaque model. Cross-sectional T1-weighted images were captured at various time points subsequent to intravenous administration of the Fe_3_O_4_-Cy probe, utilizing the respiratory gating technique. As illustrated in Figure 4A, the signals emanating from the plaque site within the aortic wall exhibited a gradual augmentation over time. The T1 images consistently portrayed an escalating bright signal in the aortic wall as time progressed. Quantitative assessment of the region of interest was conducted, unveiling an incremental trend in the signal ratio of the aorta to the surrounding muscle tissue, ultimately reaching its maximum at 48 h post-injection (Figure 4B). Consequently, we can deduce that Fe_3_O_4_ nanoparticles accumulated at the plaque site, enabling comprehensive plaque imaging and dynamic assessment of their long-term behavior.

To validate the imaging results of the plaques from the retention of Fe_3_O_4_ nanoparticles and to further elucidate the enrichment and retention of the probe at the aortic plaque site, aortic tissue was harvested and analyzed at varying intervals after the probe’s intravenous administration. The morphology and structure of the plaques were examined through H&E staining, while the retention of iron at the plaque sites was ascertained through Prussian blue staining. As evidenced in Figure 4C, the H&E staining outcomes unveiled luminal narrowing alongside pronounced accumulations of macrophages and foam cells. Prussian blue staining of aortic tissues at different time points exhibited visible positive staining at 24 h, indicating a modest degree of Fe_3_O_4_ nanoparticle enrichment. In contrast, the proportion of positive staining markedly increased at 48 h, signifying a pronounced augmentation in iron enrichment. These outcomes further bolstered the affirmation that the iron oxide nanoprobe could indeed be enriched and retained at the lesion site, thereby facilitating effective plaque imaging and dynamic assessment.

### 3.7 *In vivo* safety assessment

The preceding studies have shown the remarkable MRI efficacy of ultrasmall iron oxide nanoparticles at the site of plaques. However, ensuring their *in vivo* safety is of paramount importance. To assess the probe’s safety profile, we subjected vital organs such as the heart, liver, spleen, lung, and kidney to H&E staining at 72 h following probe administration. The H&E evaluations yielded noteworthy outcomes, as there were no discernible instances of structural impairment or pathological indications of inflammation, cellular edema, or necrosis within the examined tissues. These findings conclusively establish that our Fe_3_O_4_-Cy probe maintains exceptional biosafety levels at a dosage of 0.1 mmol/kg.

## 4 Conclusion

In conclusion, we successfully synthesized ultrasmall iron oxide nanoparticles using a high-temperature thermal decomposition method. By implementing ligand exchange and amidation reactions, we developed a dual-modality MRI/fluorescence nanoprobe based on these nanoparticles. Through comprehensive *in vivo* experiments using an atherosclerotic plaque model, we demonstrated the nanoprobe’s ability to provide an accurate morphological assessment of plaque stenosis. Moreover, the probe enables dynamic monitoring and evaluation of aortic and carotid plaques over extended periods. These results demonstrate the potential of ultrasmall iron oxide nanoparticles for prolonged observation and assessment of atherosclerosis. In clinical practice, diagnostic imaging stands as a powerful tool, furnishing doctors with comprehensive information crucial for devising effective treatment plans. Among the various imaging modalities, MRI has received considerable attention due to its high SNR, impressive spatial resolution, avoidance of ionizing radiation, and extended imaging window. Our PEGlated ultrasmall iron oxide nanoparticles exhibit excellent biocompatibility, robust magnetic properties, ease of surface modification, and passive targeting capabilities. These characteristics render them highly suitable for clinical diagnosis across a various diseases. Overall, these findings not only validate the effectiveness of ultrasmall iron oxide nanoparticles as a powerful tool for atherosclerotic plaque imaging but also lay a solid foundation for their potential clinical translation as MRI contrast agents. Our work contributes to the advancement of noninvasive diagnostic methods and holds promise for future applications in clinical practice.

## Data Availability

The original contributions presented in the study are included in the article/Supplementary Material, further inquiries can be directed to the corresponding authors.
