# Supplementary material for: Exploring atherosclerosis imaging with contrast-enhanced MRI using PEGylated ultrasmall iron oxide nanoparticles
Source: Front Bioeng Biotechnol. 2023 Sep 20;11:1279446. doi: 10.3389/fbioe.2023.1279446 (PMC10557075; doi:10.3389/fbioe.2023.1279446)
Supplement: Supplementary file 1 [file DataSheet1.docx]

Supplementary Material

Exploring Atherosclerosis Imaging with Contrast-Enhanced MRI using PEGylated Ultra-Small Iron Oxide Nanoparticles


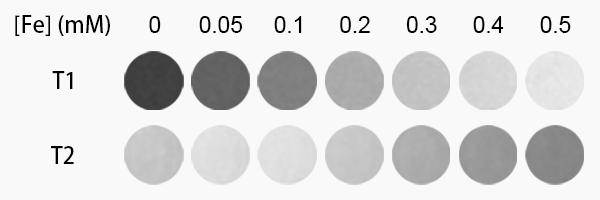


**Figure S1.** The MR images of Fe_3_O_4_-Cy at different Fe concentrations (0, 0.05, 0.1, 0.2, 0.3, 0.4, 0.5 mM).


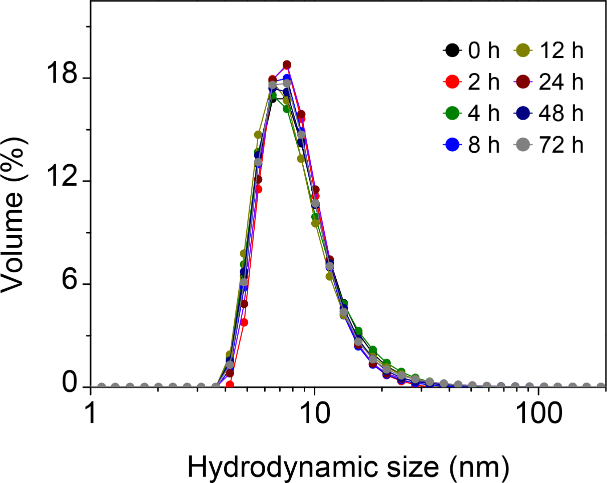


**Figure S2.** The Hydrodynamic size distribution of Fe_3_O_4_-Cy incubated with 10% FBS for 72 h.


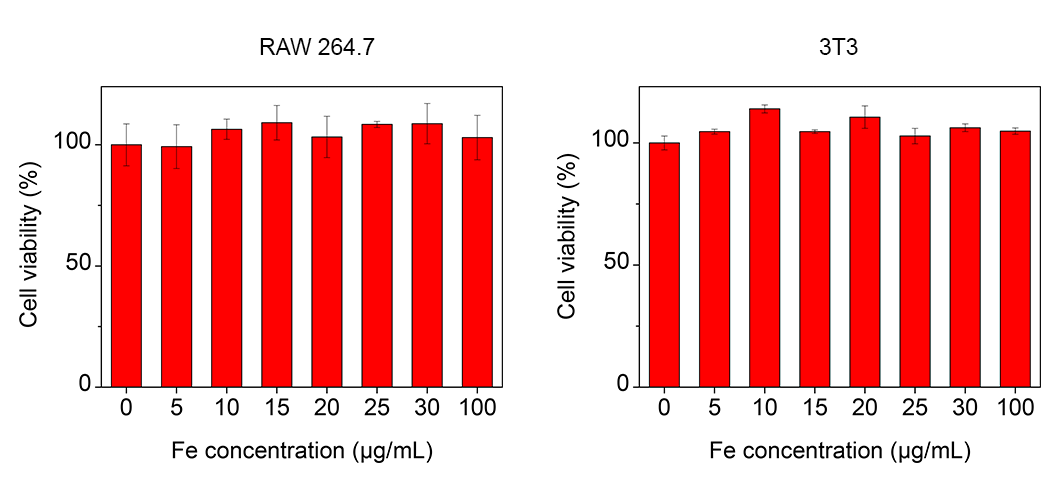


**Figure S3.** The cell viabilities of 3T3 cells and RAW 264.7 cells after incubating with Fe_3_O_4_-Cy for 24 h.


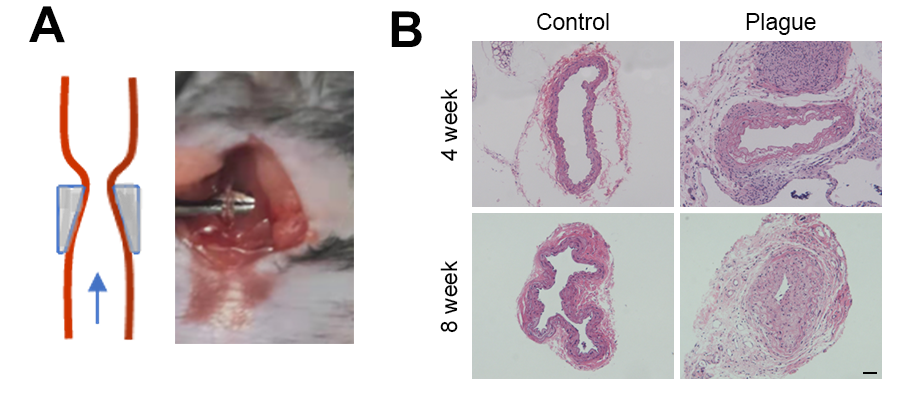


**Figure S4.** (A)The schematic illustration of the establishment of the atherosclerotic plagues; (B) H&E staining of control and plagues region at 4 week and 8 week after modeling.


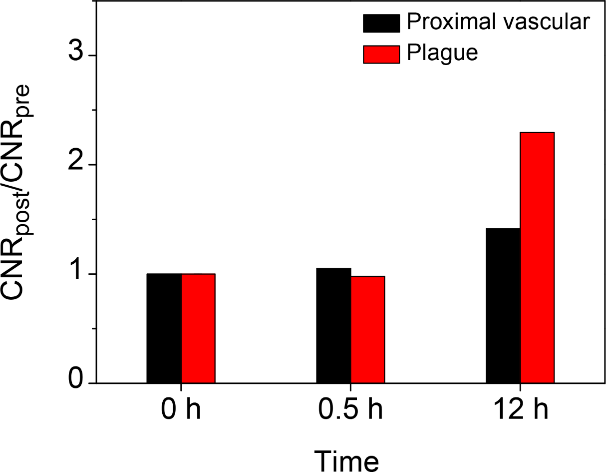


**Figure S5.** The changes of relative signal-to-noise ratio values at the proximal vascular and the plague site over time after intravenous injection of the Fe_3_O_4_-Cy.


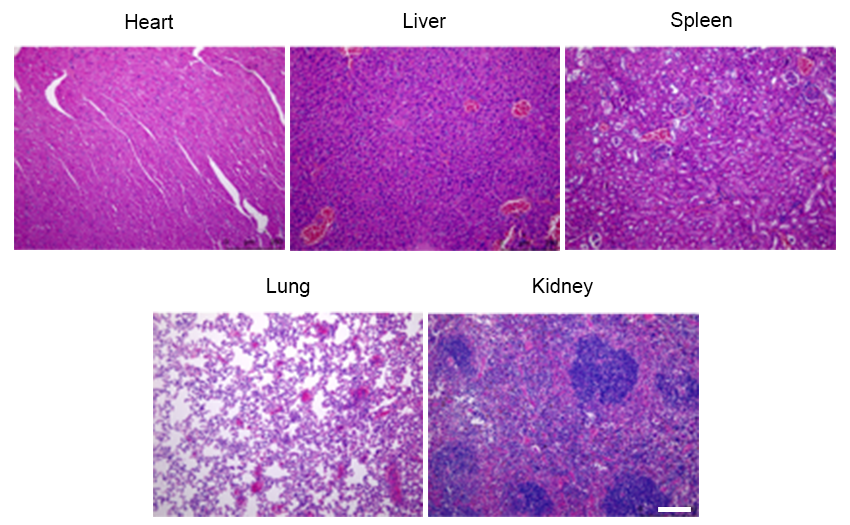


**Figure S6.** H&E staining of major organs (heart, liver, spleen, lung, kidney) extracted from mice at 72 h after injection of Fe_3_O_4_-Cy. (Scale bar: 100 μm)
